# Physical-Chemical Study of Anthracene Selective Oxidation by a Fe(III)-Phenylporhyrin Derivative

**DOI:** 10.3390/ijms21010353

**Published:** 2020-01-05

**Authors:** Carlos Diaz-Uribe, William Vallejo, Cesar Quiñones

**Affiliations:** 1 Grupo de Fotoquímica y Fotobiología, Universidad del Atlántico, Puerto Colombia 81007, Colombia; carlosdiaz@mail.uniatlantico.edu.co; 2 Facultad de Ingeniería Diseño e Innovación, Politécnico Grancolombiano, Bogotá 110231, Colombia; caquinones@poligran.edu.co

**Keywords:** sensitizer, porphyrin, hydroxyl radical, polycyclic aromatic hydrocarbons, anthracene

## Abstract

In this work, we studied the anthracene oxidation by hydroxyl radicals. Hydroxyl radical was generated by reaction of *5,10,15,20-tetrakis(4-carboxyphenyl)porphyrin Fe (III)* (TPPFe) with hydrogen peroxide under visible radiation at a nitrogen atmosphere. The TPPFe was synthesized by Adler Method followed by metal complexation with Fe (III) chloride hexahydrate. Hydroxyl radical was detected by fluorescence emission spectroscopy and we studied kinetic of anthracene selective oxidation by hydroxyl radicals through the differential method. The TPPFe was characterized by UV-Vis spectrophotometry, Dynamic Light Scattering (DLS) and Scanning Electron Microscopy (SEM) measurements. The results indicated that TPPFE was compound by micro-particles with a size distribution of around 2500 nm. Kinetic results showed that the apparent rate constant for the oxidation of anthracene increased exponentially on as temperature increases, furthermore, the activation energy for the Anthracene oxidation by hydroxyl radicals under visible irradiation was 51.3 kJ/mol. Finally, anthraquinone was the main byproduct generated after oxidation of anthracene by TPP-Fe under visible irradiation.

## 1. Introduction

Nowadays polycyclic aromatic hydrocarbons (PAH) originate from the incomplete combustion of fossil fuels are an important researching subject, from the environmental point of view, PAH are very important since they are involved in metabolic processes that affect human health [1,2]. Reactive oxygen species (ROS) are an emerging class of endogenous, highly reactive, oxygen molecules, these species can originate oxidation reactions of PAH, main ROS involve singlet oxygen and oxygen free radicals such as hydroxyl radical (HO^•^), superoxide anion (O_2_^−•^), hydroperoxyl (HOO^•^) or other similar radicals [3,4]. Specific photodynamic oxidation reactions have become an alternative methodology for PAH oxidation; the complex primary photochemical reaction processes due to an interaction of a photosensitizer with visible light could generate several reactive oxygen species in the reaction medium (^1^O_2_, O^•^, HO^•^) [5,6]. Hydroxyl radical can be achieved through different methodologies: (a) chemical trapping, (b) laser flash photolysis, (c) pulse radiolysis, and (d) laser-induced fluorescence. Hydroxyl radical reaction to PHS (e.g., Naphthalene, Biphenyl, Anthracene) is reported for ROS detection in solution [7]. F. Goulay et al., reported the first direct measurement of the reaction rate constant of a polycyclic aromatic hydrocarbon in the gas phase in the temperature range 58–470 K [8].

Different factors involve in ROS generation: photo-sensitizers, electromagnetic radiation and the molecular oxygen ^3^O_2_. Under visible light irradiation, the photosensitizer (PS) excites from basal state (^o^PS) to singlet state (^1^PS*) after that, ^1^PS* could decay to triplet state (^3^PS*) through internal cross-system process; in this stage ^3^PS* could produce radical species as hydroxyl or superoxide radicals through electron transfer reaction, this kind of reaction is known as type I [9]. Many compounds have been reported as sensitizers (e.g., porphyrin, phthalocyanines, and their metal derivatives), among these, porphyrins their metalcomplexes are macrocyclic molecules with an extensive delocalized electrons system, they have electronic and optical properties, applied in fields like artificial photodynamic therapy, solar cells and photocatalysis [10,11,12], furthermore, metalloporphyrin derivatives have reported as hydroxyl radical generators, however, knowledge about kinetic information is few; about this topic, a detailed knowledge of oxidation mechanisms initiated by molecular oxygen and OH^•^ radicals is important to optimization of experimental conditions [13]. It is known that the efficient photo-activity of both the simple porphyrins and the metal complexes porphyrins is due to their capability to produce either superoxide anion radical or singlet oxygen by type I and type II processes, respectively; for which the type II process is thought to be dominant in free base porphyrin [14]. Selective oxidation of anthracene to anthraquinone before by reaction with acetic acid [15] and metachloroperbenzoic acid by electron-withdrawing metalloporphyrins [16] has been reported.

**Scheme 1:**
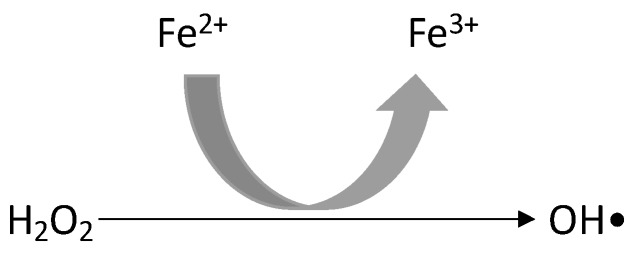
OH^•^ generation by Fenton reaction.

Considering the adverse effects on environments and human health of PAH, we selected anthracene as the target in this work because of the anthracene is considering one of the 16 priority PAHs indicated by the US environmental protection agency (EPA) [17]. There are reports concerning to oxidation of anthracene with free radicals NO_3_^•^, OH^•^ and SO_4_^•^^−^ [18,19,20,21]. Furthermore, the formation of nitro-anthracene from the degradation of anthracene by the OH^•^ and NO_3_^•^ has been investigated using quantum chemical calculations [22,23]. Karan et al. reported the photocatalytic degradation of anthracene under UV irradiation on TiO_2_ nanoparticles, they found that the main product of oxidation of Anthracene is 9,10-Anthraquinone, which is safer for the environment than Anthracene [24]. Kozak et al. removed PAHs from the pretreated coking wastewater by using CaO_2_, Fenton reagent under UV irradiation [25]. The applications of Fenton oxidation of different PAH have been shown to have great potential (e.g., photo-Fenton, sono-Fenton and electro-Fenton [26,27,28,29]). Fenton reaction mechanism relies on OH^•^ generation by the reaction of a ferrous ion with hydrogen peroxide (H_2_O_2_) this mechanism is useful to break down a different organic compound. The Scheme 1 shows the general reaction for Fenton reaction.

The photo Fenton reaction has been already used to degrade samples of phenol [30,31]. The rate of this reaction could be increased when exposed to UV light. Otherwise, although the developments related to PAH photooxidation under UV irradiation research have been significant, great attention in the field is directed to reach the PAH photooxidation under solar irradiation (visible light) to achieve a practical application [32,33].

In this work, we presented a kinetic study of the anthracene oxidation trough *5,10,15,20-tetrakis(4-carboxyphenyl)porphyrin Fe (III)* under visible irradiation, we used the initial rates method to determine activation energy and kinetic parameters of the process.

## 2. Results and Discussion

### 2.1. UV-Vis Assay

**Figure 1:**
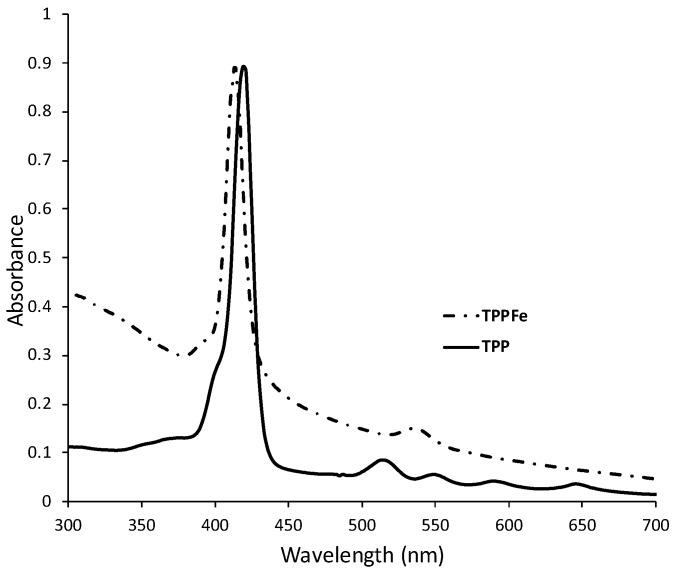
The UV-Vis spectra for the TPP and TPPFe.

Figure 1 shows the UV-Vis spectra of TPPFe and the TPP in ethanol solution. Results show the typical Soret and four Q bands for the TPP, see Figure 1. The highest band located at 419 nm was assigned to Soret band, and bands located between 516 nm and 650 nm corresponding to Q bands, these signals are associated to a_2u_(π)-e_g_^*^(π) electronic excitations. The TPPFe UV-vis spectra exhibited one Soret band (413 nm) and only a Q band (535 nm), this result is typical of this kind of compound, after metal complexing symmetry of porphyrin increases thus Q bands number decreases. Figure 1 shows little blue shift to Soret band of TPPFe, this result indicates that energy necessary for electronic transitions increases after metal complexing, this could be associated to a decreasing of the average electron density of the metalloporphyrin [34,35,36].

### 2.2. Dynamic Light Scattering Characterization

**Figure 2:**
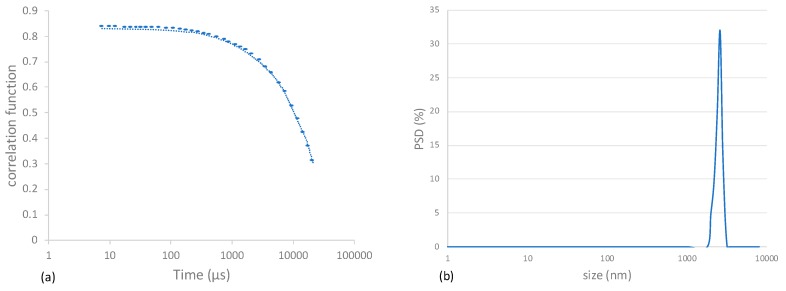
Results for DLS assay: (**a**) correlation function and (**b**) Intensity weight particle size distribution TPPFe obtained from correlation function.

Figure 2 shows the DLS assay results for TPPFe after. Figure 2a shows the correlation function (fitting quality for the diameter of the particles in suspension). Figure 2b shows the intensity weighted particle size distribution for TPPFe, the figure shows only one signal indicating TPPFe is formed by one particle type with an average size distribution of around 2500 nm. Figure 2 did not show additional signals suggesting that porphyrin aggregation was not present inside suspension under experimental conditions [37,38,39].

### 2.3. SEM Characterization

**Figure 3:**
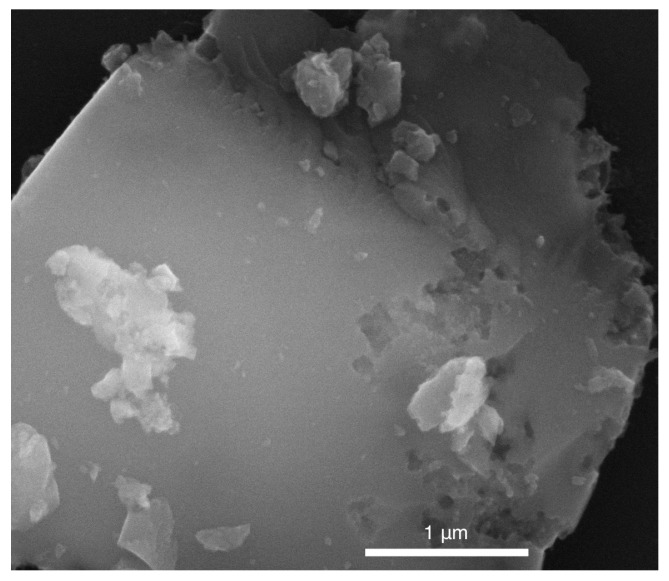
SEM micrograph for TPPFe, magnification 80 K.

The Figure 3 shows the SEM image for TPPFe powders. According to DLS results, the main size of the particles is around 2.5 μm, however, the Figure 3 shows that samples are formed by micro-particles and (Figure 3), the micro-particles show little aggregates on the surface around 300–400 nm, besides, Figure 3 shows that TPPFe surface was regular and uniform.

### 2.4. Identification of Hydroxyl Radical

**Figure 4:**
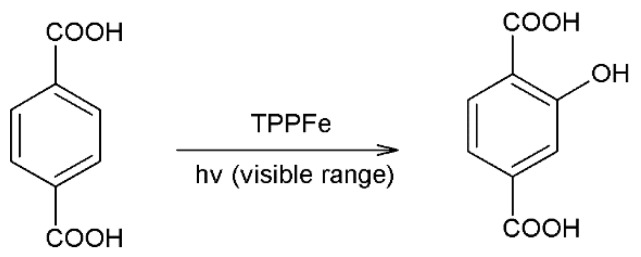
Specific oxidation chemical reaction of terephthalic acid by hydroxyl radical generated after visible irradiation of TPPFe.

**Figure 5:**
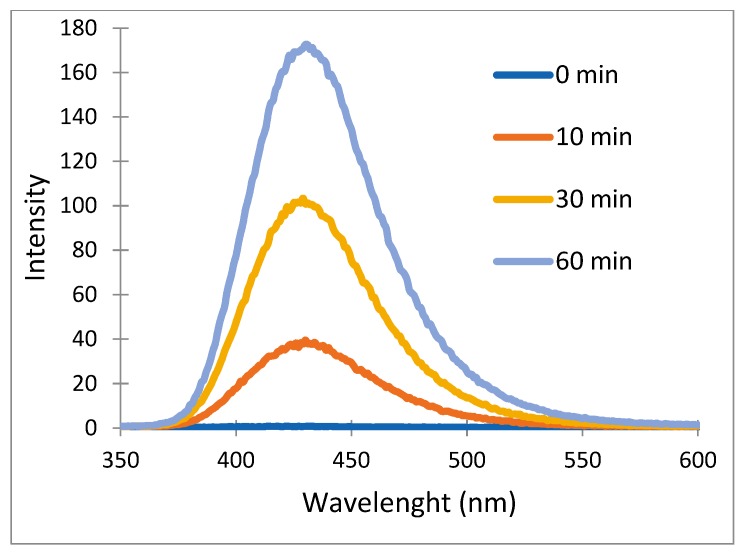
The fluorescence emission spectra of the product obtained for the reaction of hydroxyl radical and disodium terephthalate recorded at different irradiation times.

Hydroxyl radical detection was performed by selective reaction of disodium terephthalate with hydroxyl radical to form 2-hydroxyterephthalate, the Figure 4 shows the chemical reaction and the Figure 5 shows the fluorescence emission spectra of by product obtained for the reaction of hydroxyl radical and disodium terephthalate recorded at different reaction times. Results shows an increase of the maximum emission band of the 2-hydroxyterephthalic acid during the reaction time.

Due to the fluorescent product (2- hydroxyterephthalic acid) formed during irradiation are generated due to the specific reaction between hydroxyl radicals and terephthalic acid, these results corroborate that hydroxyl radicals were generated by the TCPPFe under visible light irradiation according to reaction in Figure 4 [40].

### 2.5. Kinetic Study

We determined the initial rates of reaction through the differential method for each of the experiments for determining anthracene concentration. The determination of the slopes was solved numerically using finite differences. Taking into account that the derivative of the concentration with respect to time is defined by:

(1) $$\frac{dC}{dt}=\underset{h\to \infty }{\lim}\frac{C\left(t+h\right)-C\left(t\right)}{h}$$

where *C* represents de concentration, *t* the time and *h* is the spacing constant. Because of *h* is a fixed value near to zero then, Equation (1) can be rewritten as:

(2) $$\frac{dC}{dt}=\frac{\Delta \left[C\right]\left(t\right)}{h}$$

The difference can be considered a differential operator, which matches the function *C* with Δ*C* and, by using the Taylor theorem:

(3) $$\Delta =hD+\frac{1}{2}\;h^{2}D^{2}+\;\frac{1}{3}\;h^{3}D^{3}+\ldots =e^{hD}-1\;$$

where *D* is the operator derivative, which matches *C* with its derivative (*C*’) finally, by solving the exponential:

(4) $$hD=\log\left(1+\Delta \right)=\Delta -\frac{1}{2}\;\Delta^{2}+\;\frac{1}{3}\;\Delta^{3}+\ldots$$

The first two terms of the series lead to:

(5) $$\frac{dC}{dt}=\;-\frac{C\left(t+2h\right)-4C\left(t+h\right)+3C\left(t\right)}{2h}$$

for using the first three points (*t*_0_, *C*_0_), (*t*_1_, *c*_1_), (*t*_2_, *C*_2_) whose abscissae equidistant *h* (*h* = *t*_2_ − *t*_1_ = *t*_1_ − *t*_0_ = 5 min) then, we can obtain:

(6) $$initial\;rate\left(v\right)=\;\frac{C_{2}-4C_{1}+3C_{0}}{2h}$$

**Figure 6:**
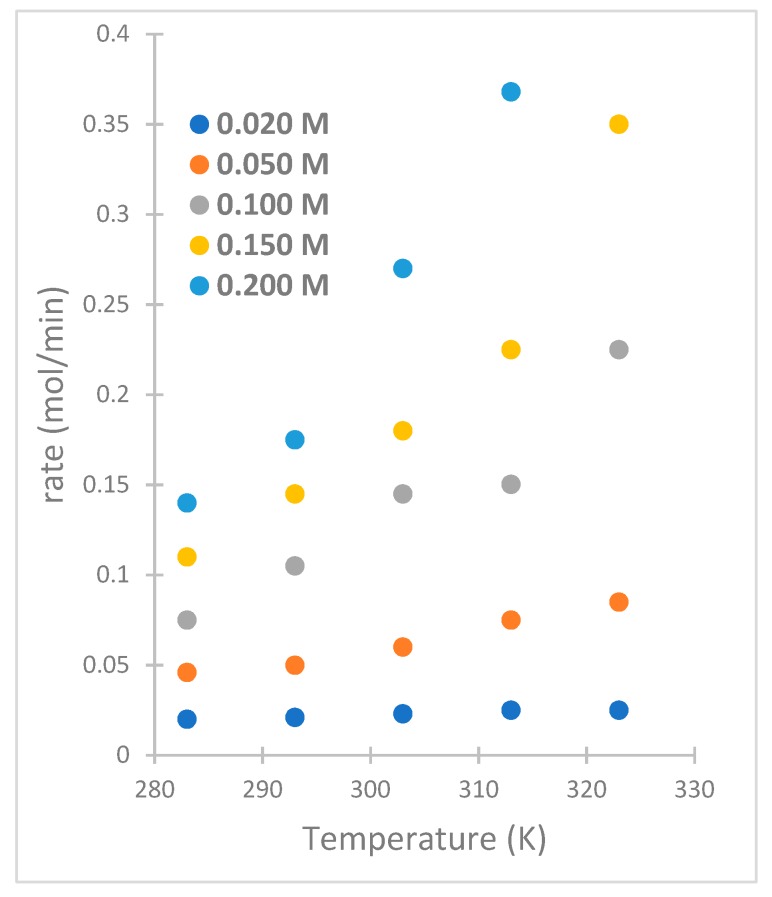
Initial rates for the oxidation of anthracene at different temperatures and anthracene concentration.

Using the experimental data and applying Equation (7), we can obtain the initial rates at different temperatures for the reactions with hydroxyl radicals [41]. The initial rates for the oxidation of anthracene by hydroxyl radical as a function of the concentration and temperature are shown in Figure 6.

The results show that the initial rates increase as the concentration of anthracene increases due to a higher probability of collision between anthracene molecules and the oxygen species present in the solution. Furthermore, Figure 6 shows that the initial rate increases with the temperature; however, no significant change is observed in the initial rate to very low concentrations of reagent. Under the experimental conditions of this study, the following rate law equation could be considered:

(7) $$r=\;k[Anthracene{]}^{\alpha }[{\mathrm{OH}}^{*}{]}^{\alpha }$$

where *r* is the initial rate as function of the reagent concentration, α and β are the reaction orders from each reagent and *k* is the rate constant. We can determinate the values of α, β in order to analyze how the initial rate depends on the initial concentrations of reagent through of initial rates method. If the initial rate depends only on the reagent concentration which is being varied [Anthracene] and if the concentration of the oxidant is in excess, we can express Equation (8) as follows:

(8) $$r=\;k_{app}{\left[Anthracene\right]}^{\alpha }$$

where *k_app_* is the apparent constant representing the product k*[HO•]^β^. If the anthracene concentration is changed, the reaction order α and the *k* value can be obtained from a linear fit of the ln *v* vs. ln [Anthracene]. Kinetic results indicated that the reaction order α for the oxidation reaction between the anthracene and the hydroxyl radical is approximately 1.0.

**Figure 7:**
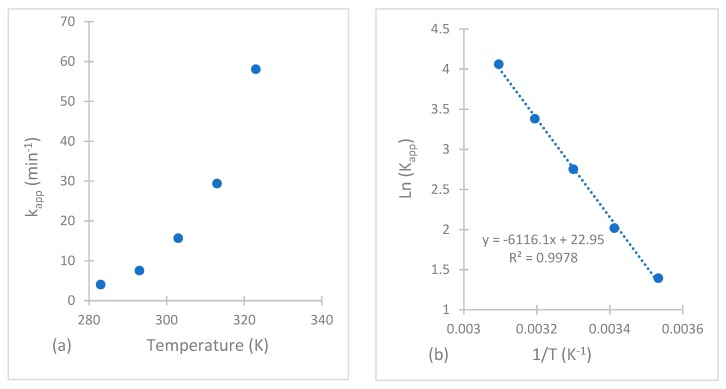
(**a**) Apparent rate constants *k_app_* as function of temperature. (**b**) *Ln* v vs.1/*T* fitting.

Figure 7a shows the *k_app_* values for oxidation of anthracene at different temperatures. Results show that the apparent rate constants increase with temperature, this result indicates that as temperatures increase more particles acquired enough energy to overpass activation energy of the process and rate constant increases also. The activation energy can be determined by the Arrhenius equation [41]:

(9) $$k_{app\left(T\right)}=Ae^{\frac{E_{a}}{RT}}$$

where *k_app_*_(T)_ is the apparent rate of the reaction (temperature dependent), *A* is a constant related with the initial reagent concentration, *E_a_* is the activation energy, and *R* is the molar gas constant. We can rewrite the Equation (9) as follows:

(10) $$Ln(k_{app\left(T\right)})=LnA-\;\frac{E_{a}}{R}\left(\frac{1}{T}\right)$$

The activation energy can be calculated from the slope of the linear fit of the natural logarithm of the apparent constant as function of 1/T. Figure 7a shows the graphic *k_app_* vs. Temperature for anthracene oxidation, and Figure 7b shows *Ln v* vs. 1/*T* fitting. The value of *E_a_* obtained for oxidation of anthracene by hydroxyl radical is 51.3 kJ/mol.

### 2.6. Analysis of the Products of Oxidation

**Figure 8:**
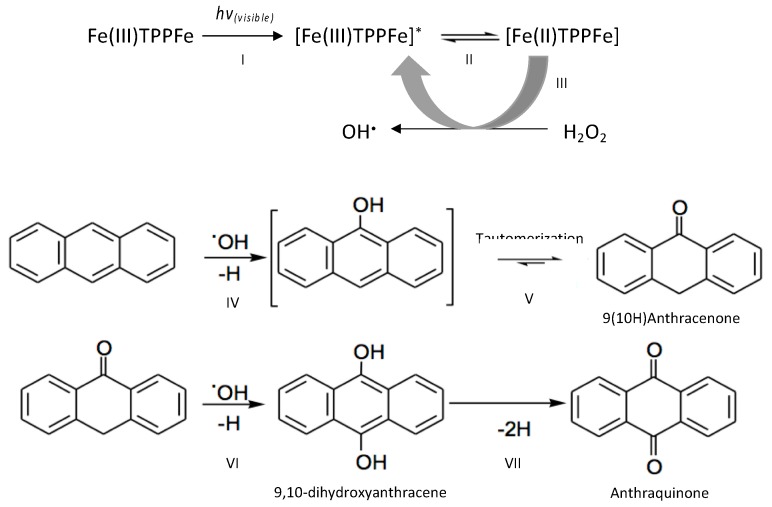
Mechanism for anthracene oxidation by hydroxyl radical: TCPPFe (**III**) absorbs radiation in visible region and then an electron is transferred from the porphyrin to Fe^3+^ (**I**). The metal is reduced from Fe^3+^ to Fe^2+^ (**II**), the Fe^2+^ interacts with H_2_O_2_ which produces hydroxyl radicals (**III**). Afterward, the hydroxyl radicals react with anthracene generating 9-hydroxyanthracene (**IV**), the tautumeration equilibrium is reached generating 9(10H)anthracenone (**V**). The subsequent addition of hydroxyl radical to yields 9,10-dihydroxyanthracene (**VI**) which can be readily oxidized to anthraquinone (**VII**).

After photooxidation process, the anthraquinone was identified by CG-ME as the only one product for anthracene reaction. In the presence of hydroxyl radical, the anthraquinone formation could be explained via the formation of 9(10H)anthracenone. The latter could be obtained by a hydrogen atom abstraction reaction following by subsequent addition of hydroxyl radical to yields 9,10-dihydroxyanthracene which can be readily oxidized to anthraquinone. These primary step events could be considered as the rate-determining the stage of the process, this mechanism is an alternate route to the mechanism proposed to anthracene oxidation in the advanced oxidation process [42,43,44]. Figure 8 proposes a mechanism leading to the formation of 9,10-anthraquinone.

## 3. Materials and Methods

### 3.1. Synthesis and Characterization

**Scheme 2:**
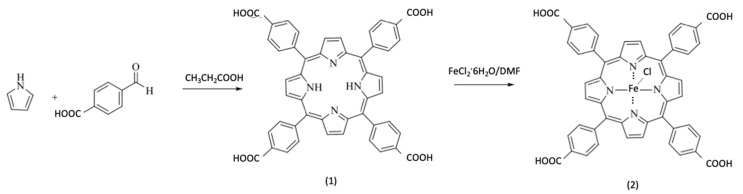
The synthesis procedure: 5,10,15,20-tetrakis(4-carboxyphenyl) porphyrin (1) and 5,10,15,20-tetrakis(4-carboxyphenyl) porphyrin Fe (III) (2).

All reagents used in this work were analytical grade, the UV-Vis spectra were taken in a Hewlett-Packard 8453 spectrophotometer. FT-IR spectra (KBr) were recorded in a Bruker Tensor 27 spectrometer, in this assay, we dissolved 2.0 × 10^−5^ g of each compound in ethyl acetate. The NMR ^1^H spectra were performed in a Bruker AC-400 spectrometer using DMSO-*d_6_* as solvent. The ^1^H MNR chemical shifts were reported as ppm (δ), relative to CDCl_3_ (signal located at 7.28 ppm). Mass spectrometry assay was measured in ESI (LC)-MS/MS ion Trap Amazon, Bruker spectrometer. The Scheme 2 shows the synthesis procedure.

*5,10,15,20-tetrakis(4-carboxyphenyl) porphyrin* (1) was synthesized by mixing pyrrole and 4-carboxybenzaldehyde (9 mmol) in propionic acid (100 mL) and nitrobenzene (45 mL). The mixture was heated for 1h at 120 °C. After cooling and solvent removal under vacuum, the porphyrin was dissolved in 250 mL of sodium hydroxide (0.10 M) and then it was precipitated by adding hydrochloric acid (1.0 M) [45]. The obtained powder was purified using column chromatography; we used silica gel (2.5 × 24 cm) as stationary phase and petroleum ether-ethyl acetate 20:2 as mobile phase (rf. 079); yield 7501 g (33%); melting point > 300 °C; UV-Vis (ethyl acetate) 419, 516, 552, 594, 650; FT-IR (cm^−1^): O-H (3200–3500), N-H (3500 band overlapped by O-H), C=O (1687), C=C (1603), C=N (1211); ^1^H RMN (400 MHz, DMSO-*d*_6_) δ (ppm): = 7.76 (8 H, d; *J* = 7.66 Hz, 3-Ar), 7.85 (8 H, d; *J* = 7.66, 2-Ar), 8.22 (8 H, d; *J* = 7.6 Hz, Py), 8.86 (4 H, s, O-H), 12.85 (2 H, s; N-H); MS (ESI-IT), *m/z*: 791.2 [M + H]^+^.

*5,10,15,20-tetrakis(4-carboxyphenyl) porphyrin Fe (III)* (2) was synthesized by mixing (1) (0.330 mmol) and iron (III) chloride hexahydrate in 70 mL of N,N’-dimethylformamide (DMF) for 2 h in reflux system. The DMF was removed by distillation and the (2) was precipitated in water. The TCPPFe was dissolved in dissolution of solid sodium hydroxide (0.10 M) and after that, the TCPPFe precipitated by adding hydrochloric acid (1.0 M). Finally, the compound was purified by column chromatography; we used silica gel (2.5 × 24 cm) as the stationary phase and petroleum ether-ethyl acetate 5:1 as the mobile, melting point > 300 °C; UV-Vis: 413, 535.

### 3.2. Physicochemical Assay

For hydroxyl radical generation, photosensitizer was added to H_2_O_2_ solution at pH = 3.0; the mixture was introduced in a batch photo-reactor in nitrogen atmosphere under visible irradiation (100 W OSRAM halogen immersion lamp). Hydroxyl radical generation was monitored through the fluorescence emission signal of 2-hydroxyterephthalic disodium (excitation at 425 nm) of the solution in a Shimadzu RF-5301PC spectrofluorometer. The reaction products were identified in a 5890 Hewlett Packard gas chromatographer, with a 5972 mass selective detector and a HP5-MS column (30 m long and 0.25 mm internal diameter) and He was the carrier gas (1 mL/min). Sample aliquots of 1,0 μL were injected with split (1:30). The temperature program: heated at 200 °C for 5 min, then 10 °C/min up to 300 °C and kept at 300 °C for 15 min. Detector conditions were 70 eV, electronic impact, 35–400 m/z mass range, 200 V EM voltage (A-tune), 20 Hz Sweep Frequency at 230 °C. The particle size in suspension was determined by the Dynamic Light Scattering (DLS). The equipment used was a Zetasizer version 6.2 of Malvern Instruments. For the analysis, 1g/L of TCPPFe was scattered in water at pH = 3. The intensity distribution seen in the DLS program was obtained by the analysis of the correlation function by the analysis of the smaller non-negative squares. Besides, the scanning electron microscopy (SEM) characteristics for TPPFe were measured in SEM Tescan VEGA3 SB equipment under excitation energy of 5 and 1 kV. Finally, reaction rates of the anthracene oxidation were obtained by using the initial slope method. In this study, we changed the concentration of anthracene from 0.02 M to 2.0 M and the activation energies for each process was calculated at different temperatures (283–323 K).

## 4. Conclusions

In this work, we studied the kinetics of the oxidation of anthracene on ferric tetracarboxyphenylporphyrin under visible irradiation. Spectrophotometric results verified metal complexation porphyrin and DSL and SEM results indicated that TPPFe was compound by micro-particles with a narrow size 2500 nm. The corresponding rate constants and activation energy were estimated for selective anthracene oxidation. Results showed an activation energy value of 51.3 kJ/mol for anthracene oxidation by hydroxyl radical. Furthermore, the fluorescent product proved that hydroxyl radical was generated after visible irradiation of TPPFe sensitizer. Finally, anthraquinone was identified as the only product during anthracene oxidation by hydroxyl radical and we proposed a possible mechanism for the oxidation process. The anthracene removal by using TPPFe under visible irradiation is an alternative procedure to removal dangerous PAH.
